# Inherent Variability of Growth Media Impacts the Ability of *Salmonella* Typhimurium to Interact with Host Cells

**DOI:** 10.1371/journal.pone.0157043

**Published:** 2016-06-09

**Authors:** Sushmita Sridhar, Olivia Steele-Mortimer

**Affiliations:** Laboratory of Bacteriology, Rocky Mountain Labs, National Institutes of Allergy and Infectious Diseases, National Institutes of Health, Hamilton, Montana, United States of America; Robert Koch-Institute, GERMANY

## Abstract

Efficient invasion of non-phagocytic cells, such as intestinal epithelial cells, by *Salmonella* Typhimurium is dependent on the *Salmonella* Pathogenicity Island 1 (SPI-1)-encoded Type Three Secretion System. The environmental cues involved in SPI-1 induction are not well understood. *In vitro*, various conditions are used to induce SPI-1 and the invasive phenotype. Although lysogeny broth (LB) is widely used, multiple formulations exist, and variation can arise due to intrinsic differences in complex components. Minimal media are also susceptible to variation. Still, the impact of these inconsistencies on *Salmonella* virulence gene expression has not been well studied. The goal of this project is to identify growth conditions in LB and minimal medium that affect SPI-1 induction *in vitro* using both whole population and single cell analysis. Here we show, using a fluorescent reporter of the SPI-1 gene *prgH*, that growth of *Salmonella* in LB yields variable induction. Deliberate modification of media components can influence the invasive profile. Finally, we demonstrate that changes in SPI-1 inducing conditions can affect the ability of *Salmonella* to replicate intracellularly. These data indicate that the specific media growth conditions impact how the bacteria interact with host cells.

## Introduction

*Salmonella enterica* serovar Typhimurium (*Salmonella* Typhimurium) is a facultative intracellular bacterium that is a major cause of foodborne gastroenteritis in humans. In the gut, *Salmonella* Typhimurium uses the Type III Secretion System 1 (T3SS1) encoded on *Salmonella* Pathogenicity Island 1 (SPI-1) to invade epithelial cells [1,2]. T3SS are needle-like apparatus used by Gram-negative bacteria to inject/translocate bacterial effector proteins into host cells. To prime *Salmonella* for invasion, SPI-1 genes encoding proteins involved in the formation of the T3SS1 needle complex and effectors are expressed [3,4]. Invasion is also facilitated by flagella-based motility [5].

The *Salmonella* SPI-1 regulon is expressed in response to environmental cues [2,6]. Under SPI-1 inducing conditions in the laboratory, a genetically identical population of *Salmonella* Typhimurium will contain two phenotypic subsets, SPI-1^+^ and SPI-1^-^, although the relative proportions of the subsets and the level of SPI-1 induction in individual cells can vary [7]. In order to address this heterogeneity, it is necessary to do single cell analysis in addition to whole population analysis. Single cell analysis, for example using fluorescent transcriptional fusions, can provide a more accurate picture of the fraction of SPI-1^+^ bacteria in a population and has been used to study the induction of SPI-1 in a variety of conditions [7–9]. Nevertheless, the cues that trigger induction of SPI-1 and motility are not well understood, and differing growth conditions are used within the field of *Salmonella* research to yield invasive bacteria.

In the laboratory, *Salmonella* are typically grown in LB (lysogeny broth or Luria-Bertani broth), a nutritionally rich media, because of its convenience and high bacterial growth yield [10]. Most commercially available LB is animal-based, containing either tryptone (enzymatic digest of casein) or peptone (enzymatic digest of proteins) digest and yeast extract (an autodigest of *Saccharomyces cerevisiae*). Sodium chloride is usually added at either 5 g/L (LB-Lennox) or 10 g/L (LB-Miller). Variability is a recognized problem due to the ill-defined nature of the major components [11,12]. We, and others, prepare SPI-1-induced invasive *Salmonella* Typhimurium by growth in LB-Miller with shaking (aeration) to late logarithmic phase. However, despite efforts to maintain consistency to the extent possible, we have seen considerable variability in the induction of invasiveness and/or the fraction of SPI-1^+^ bacteria, and we hypothesized that this was due to inconsistency between batches or preparations of LB.

While LB is widely used in laboratories studying virulence, bacterial physiologists generally use minimal media, such as M9, to study the roles of specific nutrients. Although better defined than LB, these media often contain the poorly defined Casamino acids, an acid hydrolysis of casein to provide amino acids for growth. These are used because adding purified amino acids may be expensive and impractical for routine laboratory cultures. Here, we compared bacteria grown in LB and M9 minimal media, using both whole population and single-cell analysis of SPI-1 gene expression to assess the impact of media variability on the ability of *Salmonella* to prime for invasion. With a better population-based view of SPI-1 induction *in vitro*, we found that variation in media affects both the level of induction in single cells and the percentage of the population that is induced for SPI-1. Ultimately, these variances have a measurable effect on bacterial invasion and replication in host cells.

## Materials and Methods

### Cell culture and bacterial strains

The *Salmonella enterica* serovar Typhimurium strain SL1344 was used as WT [13]. Two isogenic strains containing the plasmid pMPMA3ΔPlac P*invF*-gfp[LVA]/R and plasmid pMPMA3ΔPlac P*prgH-gfp*[LVA]/R were used to measure the expression of SPI-1 genes *prgH* and *invF*, respectively [1,2,8]. To measure the expression of flagella, a reporter for expression of *fliC* was used with a Δ*fljB* mutation on the SL1344-background: fljBA::kan/T pMPMA3ΔPlac P*fliC-gfp*[LVA]. HeLa (human cervical adenocarcinoma, ATCC CCL-2) cells were grown in Eagle’s minimum essential medium (MEM) (Corning cellgro) supplemented with 10% (v/v) fetal bovine serum (Life Technologies), 1 mM sodium pyruvate (Corning Cellgro) and 2 mM glutamate (Corning Cellgro) at 37°C in 5% CO_2_, hereafter referred to as Complete Growth Medium (CGM).

### Bacterial growth media

LB media (Difco LB-Miller cat#244620; Sigma LB-Miller cat#L3152-1KG; US Biological LB-Miller cat#L1520-01; US Biological LB-Lennox cat#L1505-01) were prepared under the same conditions to minimize external variation.

M9 media were prepared using stock solutions of 5X M9 salts (final concentrations of 90 mM Na_2_HPO_4_ • 7H_2_O, 22 mM KH_2_PO_4_, 8.56 mM (0.5 g/L) NaCl, 18.7 mM NH_4_Cl, H_2_O), 200 mM MgSO_4_, 200 mM CaCl_2_, carbon source (1 M glucose for final concentration of 22 mM, 1 M acetate for final concentration of 30 mM, 1M succinate for final concentration of 22 mM, or 50 mM saccharate (glucarate) for final concentration of 30 mM), 1 μg/mL thiamine, and 1 mg/mL Bacto Casamino acids [1,2,7,8,14,15]. M9 medium pH was raised to 7.2 using 3 M NaOH. Casamino acids (Bacto, cat#223050; Bacto Technical cat#223120; Sigma, cat#22090-500G; Remel, cat#R451092) were dissolved in H_2_O and added independently to glucose M9 at a final concentration of 1 mg/mL. All the non-glucose M9 media contained Bacto Casamino acids. For the addition of NaCl to media, a stock solution of 5 M NaCl was added before adjusting pH to 7.0 for LB or 7.2 for M9. LB media were autoclaved, and M9 media were filtered using Nalgene Rapid-Flow Sterile Disposable Filter Units with PES membranes of 0.2 μm pore size. 150 mL bottles had a filter diameter of 50 mm; 500 mL bottles had a filter diameter of 90 mm.

### Bacterial growth conditions

Bacteria were streaked from frozen glycerol stock each week. One colony was inoculated into 2 mL of medium and grown 16–18 h (overnight) at 37°C with shaking at 230 rpm in a loosely-capped 15 mL tube. For invasion and replication assays, all overnight cultures were immediately subcultured into 10 mL of the same media in loosely-capped 125 mL Erlenmeyer flasks and incubated with shaking (230 rpm) at 37°C to late logarithmic phase. LB-grown overnight cultures were diluted 1:33 in LB and incubated for 3.5 h (OD_600_ of 3.1–4.8). Saccharate M9-grown overnight cultures were diluted 1:17 in saccharate M9 and incubated for 5.25 h (OD_600_ of 2.5–3.0). The optical density of the cultures was measured at a 1:5 dilution, in order to remain within the linear range, and then multiplied by 5.

### Promoter activity assays

Overnight cultures were diluted 1:10 in media, then immediately transferred in triplicate to a 96-well plate in 200 μL volumes. These sub-cultures were maintained shaking at 37°C in an automated plate reader (Tecan Infinite M200) with OD_600_ and GFP fluorescence readings taken every 15 min over 8 h. To calculate % promoter activity, the maximum GFP intensity achieved was divided by OD_600_ to normalize for bacterial numbers, and averaged across three experiments. For most experiments, the data was normalized to the condition with the highest promoter activity.

### Invasion and replication assays

HeLa cells were seeded at 5x10^4^ on 24-well plastic (Costar) tissue culture-treated plates 20–24 h before infection. Bacteria were grown as detailed above and a gentamicin protection assay was performed, as described previously [7–9]. Briefly, bacteria were added (T_0_) at an MOI of ~40:1 at 37°C for 10 min before being washed twice in warm 1X Hanks’ Balanced Salt Solution (HBSS) (no calcium, no magnesium, no phenol red) (Gibco) and replaced with CGM. Thirty minutes post-infection (T_30_), medium was replaced with CGM supplemented with 50 μg/mL gentamicin and 500 μg/mL histidine for 1 h. Histidine was added because SL1344 is a histidine auxotroph and there can be some restriction of intracellular growth in the absence of supplemental histidine [16]. At 1.5 h post-infection, medium was replaced with medium containing 10 μg/mL gentamicin and 500 μg/mL histidine. For invasion assays, invasion was calculated at 1.5 h post-infection. At 1.5, 4, 6 and 8 h post-infection, cells were lysed with 0.2% (w/v) sodium deoxycholate in PBS and diluted serially in PBS for plating on LB agar.

### Flow cytometry

P*prgH*-gfp bacteria grown overnight and subcultured in 10 mL LB or saccharate M9, as detailed above, were washed once by centrifugation at 8000 x g for 2 min, resuspended in HBSS, and 10 μL LB-grown or 20 μL M9-grown bacteria were fixed in 500 μL of 2.5% (w/v) paraformaldehyde (PFA) in PBS for 15 min. Bacteria were washed twice in PBS and incubated with Syto41 (Life Technologies) at 1:500 for 30 min in the dark. Stained bacteria were washed in PBS and subsequently resuspended in 1 mL PBS for analysis on a BD LSR II flow cytometer (BD Bioscience). Data were analyzed using FlowJo software (Tree Star). Samples were gated on Syto41^+^ events, and the % of GFP^+^ events was measured. Graphs of mean fluorescence intensity (MFI) were made using GraphPad Prism, and mean ± S.D. was plotted from three independent experiments.

### Statistical analysis

Statistical analyses were performed using GraphPad Prism software. All analyses were conducted on three independent experiments, and the mean ± S.D. was calculated. Statistical tests used are described in each figure legend.

## Results

### *Salmonella* SPI-1 induction is not consistent across commercial LBs

**Fig 1:**
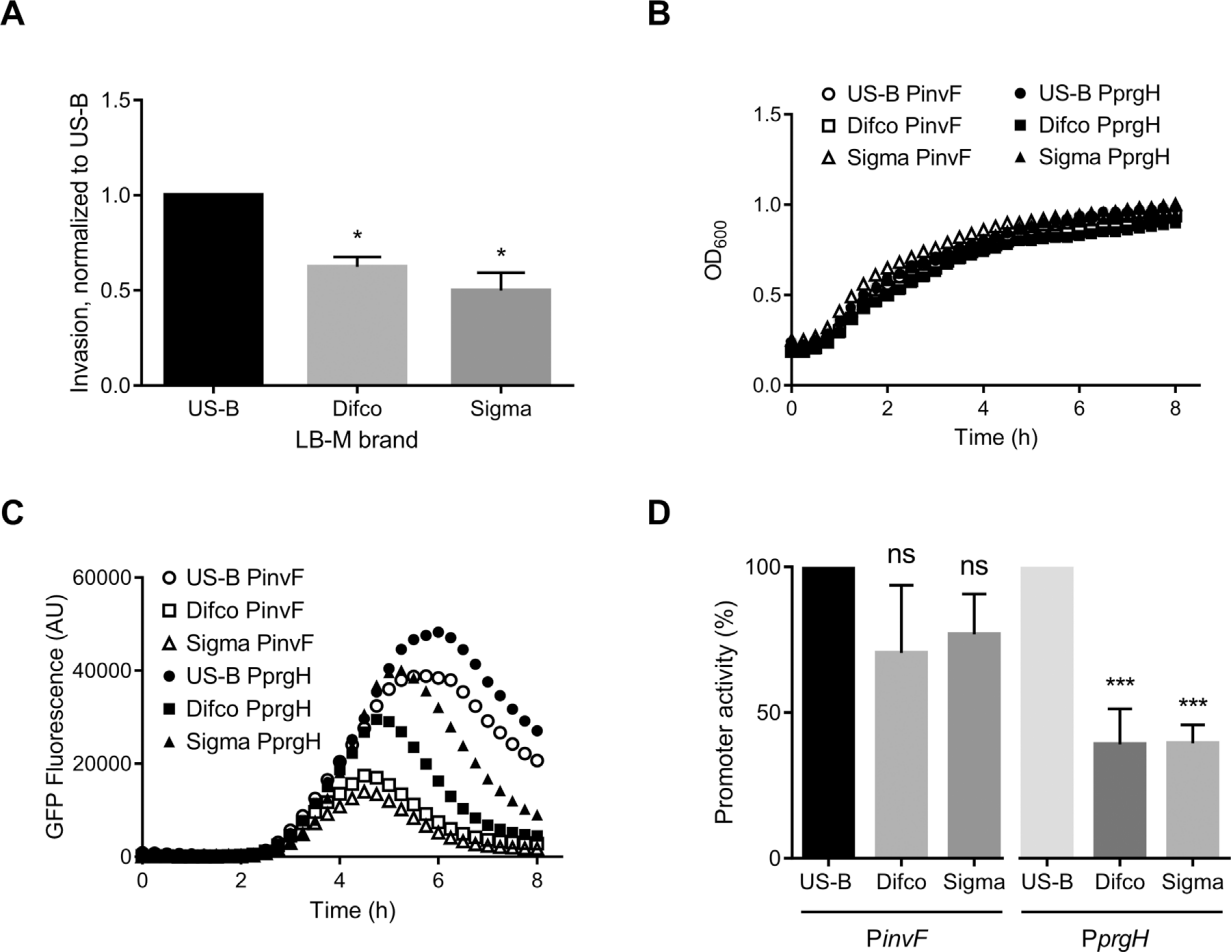
Comparison of bacterial invasiveness after grown in different brands of LB-Miller. A. Invasion assays were conducted on HeLa cells using SL1344 bacteria grown in US Biological (US-B), Difco, or Sigma LB-Miller. Data are % of the inoculum found to be intracellular at 1.5 h post-infection, normalized to US-B invasion from n = 3, showing mean ±SD. Plate reader growth curves (B) and P*prgH* activity (C) of the SPI-1 reporter strain (P*prgH*-gfp [LVA]) are shown for one representative experiment (n = 3). D. % promoter activity was calculated from n = 3 of GFP/OD600 at maximum promoter activity; normalized to US-B LB-M grown bacteria. Data are mean ±SD; statistical analyses were done using a one-way ANOVA with Dunnett’s multiple comparisons test (* *p* < 0.05).

In order to study how *Salmonella* invades epithelial cells, we routinely grow the bacteria for 16–18 h with aeration in 2 mL and then subculture in 10 mL to late-log phase, when SPI-1 is induced, before adding to HeLa cells. We have noticed substantial variation in the degree of *Salmonella* Typhimurium invasion of HeLa cells between experiments, and we hypothesized that the LB used for culturing the bacteria may be responsible. To directly compare the amount of variation between common lab sources of LBs we obtained LB-Miller powder from three sources (US Biological, Difco, and Sigma). Some manufacturers now produce animal-free or vegan formulations due to concerns about exposure to infectious diseases, so we included a vegan LB-Miller (US Biological) as one of our media. The LB media were prepared at the same time in order to minimize any variation arising from the preparation process. We used a standard gentamicin protection assay in HeLa cells, a cell line commonly used to study *Salmonella*, to assess invasiveness of bacteria grown to late-log phase. The OD_600_ after the 3.5 h subculture was 4.5, 3.1, and 4.2 for US Biological, Difco, and Sigma, respectively. We found considerable variation in invasion, which did not correlate with the OD_600_ values. Bacteria grown in US Biological LB-M were the most invasive (Fig 1A, 38% and 50% higher invasion than Difco and Sigma, respectively).

This difference was not due merely to different numbers of bacteria from each culture; as calculated from plating bacterial cultures, inoculums were similar (2.5x10^6^ ± 2.0x10^5^, 2.5x10^6^ ± 2.1x10^5^, and 3.0x10^6^ ± 3.0x10^5^ for US Biological-, Difco-, and Sigma-grown bacteria, respectively).

We subsequently compared SPI-1 induction using fluorescent reporter assays for SPI-1 genes *invF* and *prgH*, in which the promoter of either *invF* or *prgH* drives the expression of GFP[LVA], a destabilized short half-life version of GFP [8,10]. To facilitate comparison of multiple conditions over time in a plate reader, we modified our 10 mL sub-culture system used above to 200 μl in the wells of a 96-well plate. Bacteria bearing either the P*invF* or P*prgH* reporter plasmid were grown overnight as above, sub-cultured at 1:10 in a 96-well plate, then incubated at 37°C with shaking in a fluorescence plate reader. Under these conditions, growth is slower than in 10 mL cultures (late logarithmic phase was at ≈ 4.5–5 h vs ≈ 3.5 h.), but SPI-1 induction still coincided with late logarithmic phase as previously reported (Fig 1B and 1C) [7,8,11,12]. Growth, as measured by OD_600_, was similar between conditions and strains (Fig 1B).

**Fig 2:**
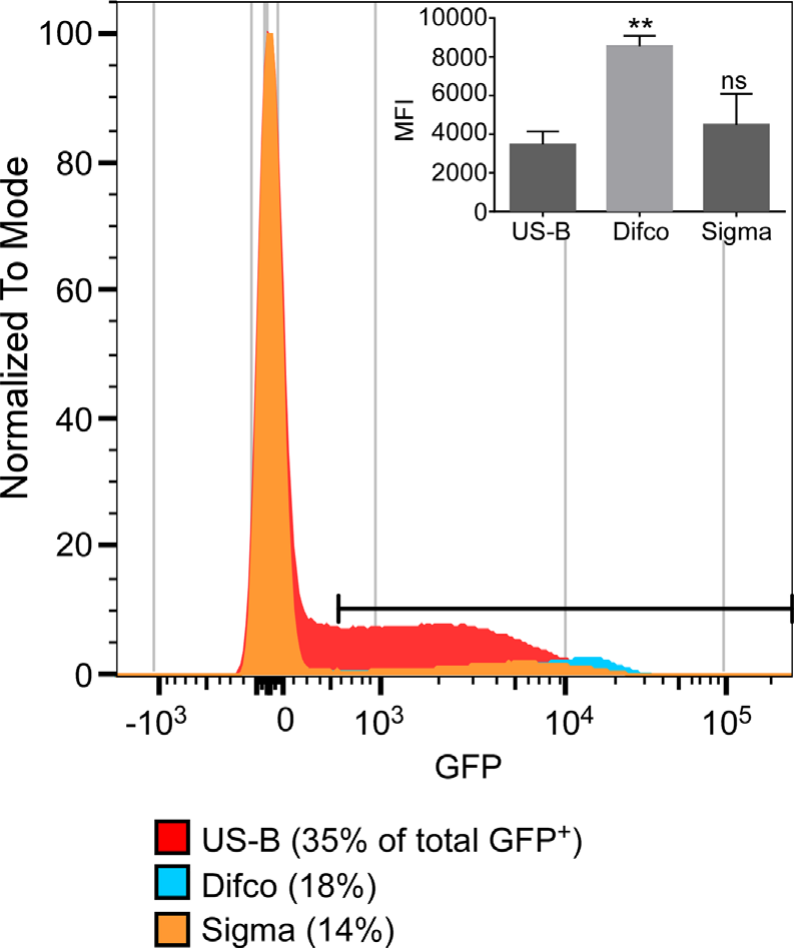
Percentage of SPI-1 induced bacteria varies across LB media. Flow cytometry data from a representative experiment showing GFP-intensity for the P*prgH* reporter strain grown in US Biological, Difco, or Sigma LB-Miller. Inset: MFI of gated GFP^+^ bacteria for the P*prgH* reporter strain. Data shown are mean ±SD, n = 3. Statistical analysis was done using a one-way ANOVA with Dunnett’s multiple comparisons test (* *p* < 0.05).

We have shown previously that SPI-1 induction peaks during late-log phase [8]. Here, P*invF* and P*prgH* expression were induced during late-log phase in all three LB media; however, there were differences in the kinetics and peak activity (Fig 1D) [11,17]. P*invF* and P*prgH* activity were highest when bacteria were grown in US Biological LB-Miller compared to Difco LB-Miller or Sigma LB-M (≈40% P*invF* and ≈75% P*prgH* activity in Difco and Sigma compared to US Biological). Plate reader assays are an efficient way to follow changes in gene expression in the total bacterial population over time, but they do not provide any information on the fraction of bacteria that are induced or whether induction levels differ between individual bacteria. To do this we used a flow cytometry approach to assess the amount of *prgH* expression in single cells upon growth to late-log phase (Fig 2). Subcultured (10 mL) bacteria at the peak of GFP expression were analyzed by flow cytometry to determine the proportion of bacteria expressing *prgH*. We found that in US Biological LB-M, ~35% of bacteria are GFP^+^, whereas only ~13% and ~17% of Difco and Sigma LB-M, respectively, are GFP^+^ (Fig 2). Interestingly, although Difco yielded the lowest percentage of GFP^+^ bacteria, the MFI of these bacteria was higher than the MFI of the GFP^+^ bacteria in the other media (Fig 2). That the GFP^+^ bacteria grown in Difco were a smaller percentage of the total than GFP^+^ bacteria grown in US Biological LB-M may have accounted for their lower invasion efficiency (Fig 1A). This further confirmed that US Biological LB-M most efficiently induced SPI-1 in the greatest number of bacteria. US Biological LB-M was used for subsequent experiments.

### Variability of SPI-1 induction in M9 minimal medium

**Fig 3:**
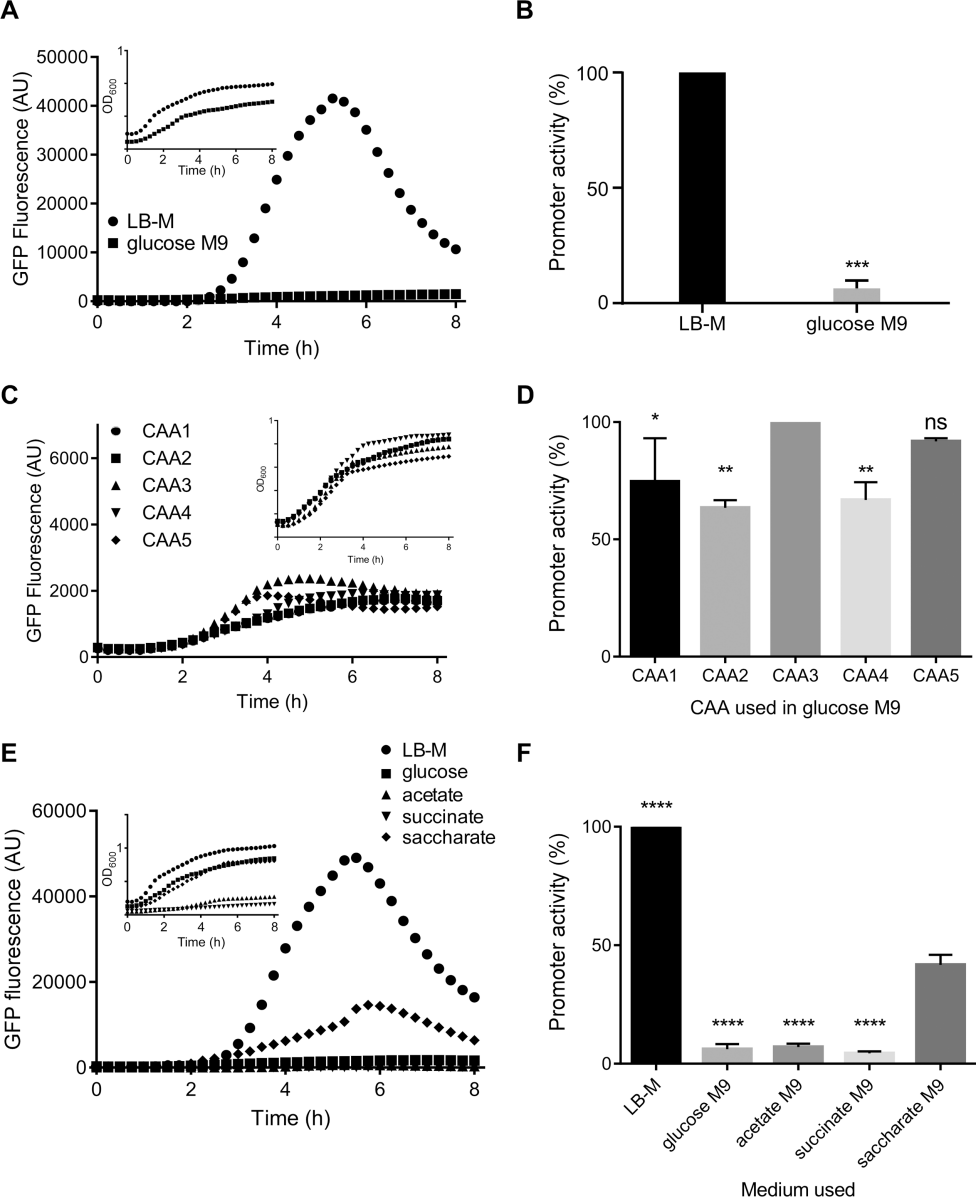
SPI-1 induction varies in M9 minimal medium. A, C, E. P*prgH* activity of the SPI-1 reporter strain (P*prgH*-gfp [LVA]) from one representative experiment. B, D, F. Comparing LB-M to glucose M9, glucose M9 containing different brands of Casamino acids, and 3 alternative carbon sources in M9, respectively. % promoter activity was calculated from n = 3 at maximum promoter activity of GFP/OD600; normalized to LB-Miller grown bacteria (B and F) or those grown with CAA3 (D). The Casamino acids used are ‘existing’ Bacto (CAA1), Bacto (CAA2), Bacto Technical (CAA3), Sigma (CAA4), Remel (CAA5). Statistical analyses were unpaired t-test with Welch’s correction (B) or a one-way ANOVA with Dunnett’s multiple comparisons test (D, F). Data shown are mean ±SD (* *p* < 0.05).

To assess SPI-1 induction in minimal media, we used M9 media supplemented with 22 mM glucose. Glucose was selected as the carbon source because it has been shown to be an important nutrient source for *Salmonella in vitro* [18]. We used the P*prgH* reporter to assess SPI-1 induction in M9 minimal medium, with glucose as the carbon source. As expected, growth is slower in glucose M9 than in LB-M (Fig 3A inset).

Strikingly, SPI-1 induction was barely detectable in glucose M9 even at peak activity (Fig 3A and 3B, 6% of activity in LB-M). M9 medium is supplemented with Casamino Acids, a component as variable as tryptone or peptone digest in LB, and we considered whether different sources of Casamino Acids in M9 would affect bacterial SPI-1 induction. We compared Casamino Acids from four commercial vendors (Bacto, Bacto Technical, Sigma, and Remel, designated CAA2, CAA3, CAA4, CAA5, respectively), in addition to our existing lot number of Bacto Casamino Acids (CAA1). Fresh glucose M9 was supplemented with each of the Casamino Acids. SPI-1 induction was then assessed as above. Similar to what we observed in LB, SPI-1 induction in M9 medium containing Casamino Acids from different sources was variable. CAA3 yielded the highest *prgH* promoter activity, which was 26% higher at peak induction than that obtained with CAA1 (Fig 3C and 3D). Thus, modulating the source of Casamino Acids can affect SPI-1 induction, and we were able to slightly increase SPI-1 induction above our baseline.

### Comparison of carbon source in M9 minimal medium

Despite the variation introduced by different Casamino Acids, SPI-1 induction in glucose M9 was very low compared to that in LB-M. Glucose is the default carbon source used in minimal medium for *Salmonella* and *E*. *coli* and is required for systemic infection of mice and interactions with HeLa cells [18,19]. Other carbon sources may be important for intestinal colonization by enteric bacteria including commensals and pathogens like *Salmonella* Typhimurium [20]. As a small sample set of possible carbon sources, we selected three additional carbon sources based on their availability in the human gut: acetate, succinate, and saccharate (glucarate). Each was supplemented into the standard M9 background containing Bacto Casamino Acids. Under these conditions, *Salmonella* did not grow well in acetate or succinate media, but growth in saccharate and glucose were very similar (Fig 3E inset).

Strikingly, P*prgH* activity was highest in the presence of saccharate, 34% higher than that of glucose M9-grown bacteria and even reaching ≈ 40% of the level observed in LB-M grown bacteria (Fig 3E and 3F). Thus, in M9 minimal medium, saccharate is a better carbon source than glucose at inducing SPI-1. Given that saccharate M9 supported both growth and SPI-1 activity, we further compared LB-M and saccharate M9.

### Effect of NaCl in media on SPI-1 induction

**Fig 4:**
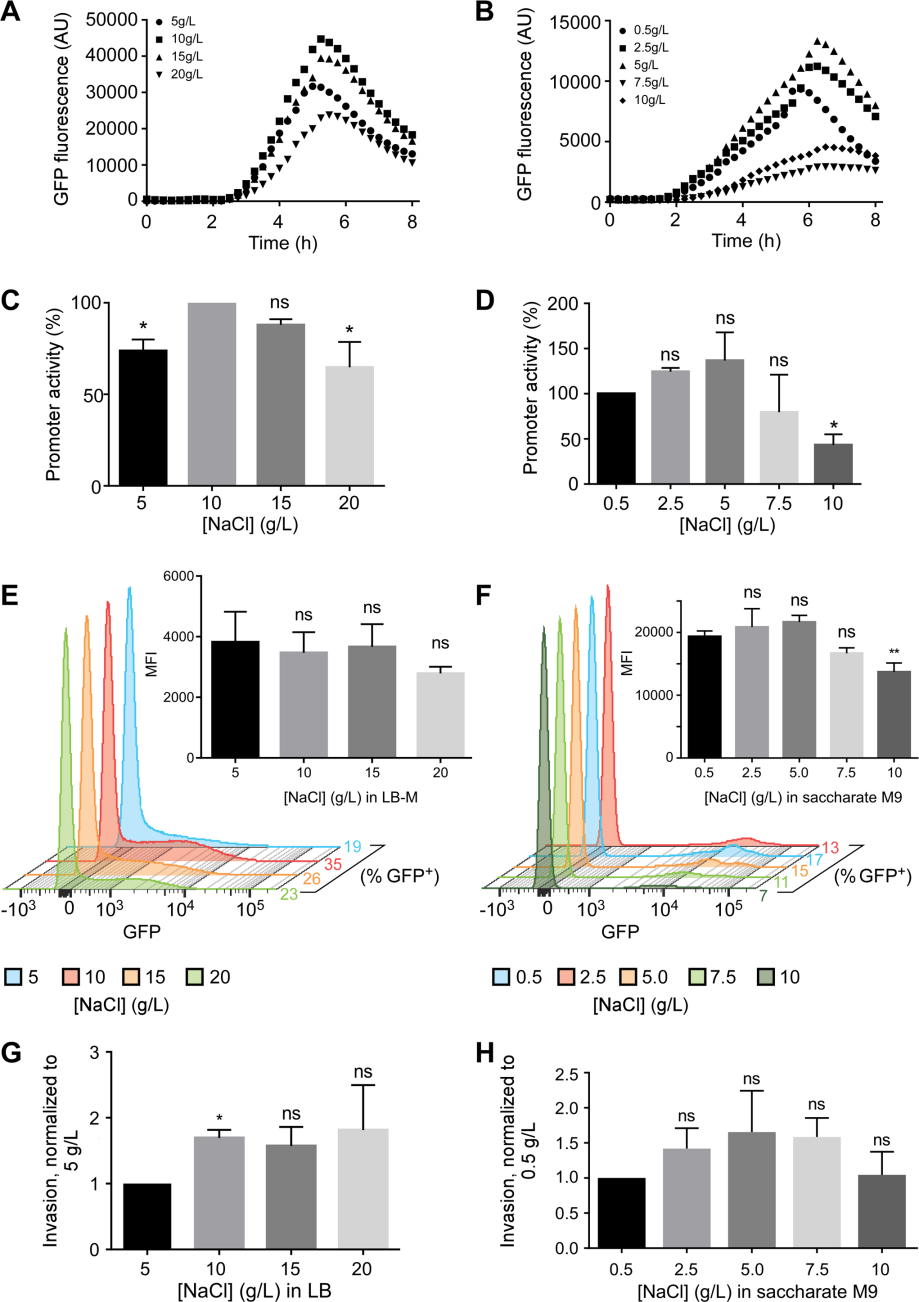
NaCl effect on SPI-1 induction and invasiveness. A, B. P*prgH* activity in bacteria grown in LB (A) or saccharate M9 (B) supplemented with the indicated amounts of NaCl, representative of three independent experiments. C, D. Peak promoter activities normalized to 5 g/L LB (C) or 0.5 g/L NaCl saccharate M9 (D). E, F. Flow cytometry from a single representative experiment showing GFP-intensity for the P*prgH* reporter strain grown in LB (E) or saccharate M9 (F) supplemented with the indicated concentrations of NaCl. The insets show MFI of gated GFP^+^ bacteria grown in each medium. G, H. Invasion of HeLa cells by SL1344wt bacteria grown in LB (G) or saccharate M9 (H). A one-way ANOVA with Dunnett’s multiple comparisons test was used for peak promoter activity, MFI, and invasion assay data. Data shown are mean ±SD of n = 3 (* *p* < 0.05).

Another component implicated in SPI-1 induction is NaCl [4]. Some labs, including ours, use LB-Miller (10 g/L NaCl) to induce invasiveness, however other formulations, such as LB-Lennox (5 g/L NaCl) are also used. To directly assess the impact of [NaCl] on SPI-1 induction, we subcultured the P*prgH* transcriptional reporter strain in LB containing 5, 10, 15 or 20 g/L NaCl. While NaCl concentration did not affect bacterial growth or the timing of *prgH* expression (maximal induction at ≈ 5 h in all cases), it did affect the level of expression. LB-Miller (10 g/L NaCl) yielded the highest P*prgH* activity (set to 100%), compared to ≈ 74% for 5 g/L, ≈ 88% for 15 g/L, and ≈ 65% for 20 g/L (Fig 4A and 4C).

Using flow cytometry, we found, consistent with the bulk promoter activity assay, that growth in LB-Miller (10 g/L) resulted in more SPI-1^+^ bacteria (35% compared to 19%, 26%, and 23% in 5 g/L, 15 g/L and 20 g/L NaCl LB, respectively). The MFI of GFP ^=^ bacteria was similar in all conditions (Fig 4E inset).

Invasion of host cells is a multi-factorial process, which may not be accurately estimated by gene expression alone. Thus, we also assessed the ability of *Salmonella* to invade HeLa cells. Using the gentamicin protection assay, we found no significant difference in the invasiveness of bacteria grown in 10 to 20 g/L NaCl, although there was a significant drop in invasion when LB-Lennox (5 g/L NaCl) was used (63% invasion compared to 10 g/L) (Fig 4G).

Because of M9 media composition, the concentration of NaCl differs significantly from that of LB-M. To study the effect of NaCl on SPI-1 induction of bacteria grown in saccharate M9 minimal medium, which contains 0.5 g/L NaCl, we added NaCl for final concentrations of 2.5 g/L, 5.0 g/L, 7.5 g/L and 10 g/L. P*prgH* activity was generally decreased at the highest concentrations of 7.5 g/L or 10 g/L (Fig 4B) although repeated experiments only revealed a significant decrease at 10 g/L (Fig 4D). Flow cytometry revealed that ≈ 8–14% of bacteria were GFP^+^ (Fig 4F). Strikingly, the MFI of the GFP^+^ *Salmonella* from saccharate M9 cultures (≈ 20,000) was >4-fold higher than those from LB cultures (≈ 4000) (Fig 4E and 4F insets). Invasion assays did not reveal any significant impact of NaCl concentration, although the trend of decreasing invasion of bacteria grown in 7.5 or 10 g/L NaCl media was consistent with P*prgH* activity from the promoter activity assay and flow cytometry (Fig 4H). Overall, these experiments suggest that, with respect to SPI-1 induction, the optimal concentration of NaCl in LB is 10 g/L (LB-Miller) and 0.5–5.0 g/L in saccharate M9.

### Growth condition differences affect intracellular replication

**Fig 5:**
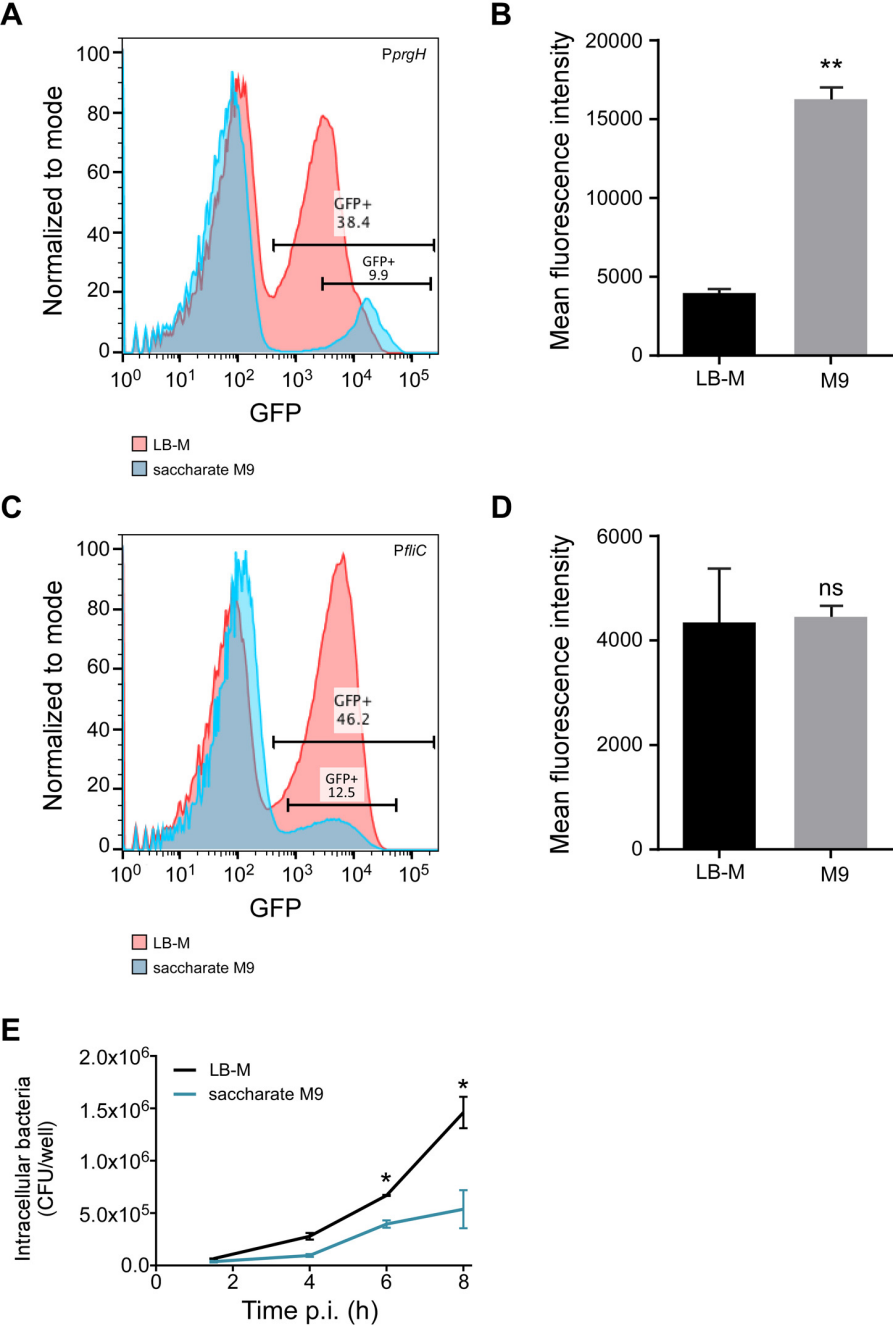
Intracellular replication of *Salmonella* Typhimurium in HeLa cells is determined by pre-invasion growth conditions. A, C. Flow cytometry from a single representative experiment showing GFP-intensity for the P*prgH* (A) and P*fliC* (C) reporter strains, grown in LB-Miller (red) or saccharate M9 (blue). B, D. MFI of gated GFP^+^ bacteria for the P*prgH* (B) and P*fliC* (D) reporter strains. Data shown are mean ±SD, n = 3 (* *p* < 0.05). E. Gentamicin protection assay showing intracellular replication of bacteria grown under SPI-1-inducing conditions in LB-M or saccharate M9. Data shown are mean ±SD, n = 3. A two-way ANOVA with Tukey’s multiple comparisons test was performed.

Next, we directly compared induction of SPI-1 in LB-Miller with saccharate M9 (0.5 g/L NaCl) using our standard SPI-1 inducing conditions. Using the P*prgH*-gfp plasmid-bearing strain for flow cytometry, we found more GFP^+^ bacteria in LB-M (≈40%) than in saccharate M9 (≈10%) (Fig 5A).

However, as noted previously [8], there was a stark difference in the MFI between the two growth conditions; the MFI of GFP^+^ *Salmonella* in saccharate M9 was > 4-fold higher than the MFI of GFP^+^ bacteria in LB-M (Fig 5B). Thus, although there were fewer SPI-1^+^ bacteria in saccharate M9 medium, these bacteria were more highly induced for *prgH* expression (Fig 5B).

Because we are interested not only in SPI-1 but more broadly the invasive phenotype of *Salmonella* grown in culture, and motility contributes to invasion, we next looked at a motility gene. Under certain conditions the flagellar regulon, including the *fliC* gene that encodes flagellin, is co-regulated with SPI-1 [21–26]. Therefore, to test whether motility and invasion are induced in the same growth conditions, we used a *fliC* transcriptional reporter, P*fliC-gfp*[LVA]. Flow cytometry analysis showed that bacteria grown in LB-M or saccharate M9 have populations of *fliC*-induced bacteria. Similar to P*prgH*, the P*fliC* GFP+ population was larger in LB; 46% in LB-M and 12% in saccharate M9, although there was no significant difference in MFI between the two conditions (Fig 5C and 5D). Overall, we found that LB-M and saccharate M9 as culture media facilitate both SPI-1 and flagellin induction, two important aspects of the invasive phenotype.

The ability of *Salmonella* to replicate within epithelial cells can be affected by the growth conditions prior to invasion [8]. Therefore we compared intracellular replication of bacteria that had been grown in LB-Miller versus saccharate M9 (Fig 5E). Bacteria grown in LB-Miller replicated to higher levels than those grown in M9, with 41% more intracellular bacteria at 6 h and 63% more at 8 h post invasion (Fig 5E). Thus, although the saccharate-grown bacteria are able to invade HeLa cells, their ability to replicate within the intracellular environment is reduced compared to as those grown in LB-Miller. Overall, we found that differences in growth conditions directly impact bacterial survival and replication in host cells.

## Discussion

SPI-1 was identified and characterized in the mid-1990s as an important factor in *Salmonella*’s ability to invade epithelial cells (for review see [27]), but laboratory conditions for induction of SPI-1 are still not standard or consistent across the field. In our lab, despite monitoring and careful control of growth conditions, we have observed that the level of SPI-1 induction fluctuates, and we hypothesized that this was due to the inherent variability of LB medium. Here, we have looked at sources of variability in SPI-1 growth conditions. We wanted to address this issue in LB and compare it to deliberately modified minimal media. For our baseline comparison, we used previously established SPI-1 inducing conditions: late-log phase growth with aeration in LB-Miller [8,28,29].

We used a fluorescent transcriptional reporter to monitor variations in SPI-1 induction at both the whole population and single-cell levels. This approach revealed that the imprecise nature of the organic constituents (eg. tryptone digest or Casamino Acids) in LB and M9 minimal medium affects SPI-1 induction. Thus, different commercial sources of LB may yield varying levels of SPI-1 induction, and media formulations should be optimized at the outset of experiments for reliable results and/or optimal SPI-1 induction. Another component in LB reported to affect SPI-1 induction is the concentration of NaCl, and we determined that there is a considerable degree of tolerance in the system for the concentration of NaCl. However, low salt content LB-Lennox (5 g/L), an LB formulation frequently used, is not optimal for SPI-1 induction.

Glucose is often the default carbon source used for growth of *Salmonella* in minimal medium; however, we found under the conditions used here that it didn’t induce SPI-1. *Salmonella* can metabolize a variety of carbon sources, including short chain fatty acids (SCFAs) such as acetate, D-saccharate, succinate, and several glucose derivatives or related compounds [30–34]. Therefore, we selected three alternative carbon sources to compare in M9: acetate, succinate, and saccharate. Saccharate was the only one that supported both growth and SPI-1 induction. Evidence of saccharate, an oxidized downstream product of glucose, as a viable sole carbon source for *Salmonella* Typhimurium comes from an early study by Gutnick *et al*., 1969 [35–38]. *Salmonella* virulence has been shown to be enhanced when saccharate catabolism occurs [39,40]. Future studies will further address the importance of carbon source in modulating SPI-1 induction.

SPI-1 has been shown to occur in a bistable manner [6,7,41]. However, one interesting observation was that depending on the media conditions, we see substantial differences in the intensity of fluorescence in individual bacteria. The single-cell analysis revealed that the saccharate M9-grown bacteria were a brighter, smaller population. In addition, the MFI profiles did not correlate between P*prgH* and P*fliC* for bacteria grown in the two media; only the P*prgH* bacteria had a significant MFI difference when grown in LB-M versus saccharate M9, suggesting that this degree of induction is specific for SPI-1.

Ultimately, we were interested in the effect of media growth conditions on intracellular survival and replication, and we confirmed our GFP reporter findings by invasion and replication assays using HeLa cells. Using LB-M and saccharate M9 to grow *Salmonella*, we found that both growth conditions support intracellular replication. In the saccharate M9 condition, there are fewer SPI-1^+^ bacteria, but they are more highly induced. There seems to be a correlation between the level of SPI-1 induction, size of the SPI-1^+^ population, and the ability to replicate intracellularly. This suggests that SPI-1 is not simply on or off; there is a finer level of control that affects intracellular replication. In fact, it has been shown that SPI-1 expression can come at a cost, and this might explain why i) there are fewer SPI-1^+^ bacteria in saccharate M9, and ii) why the SPI-1^+^ bacteria do not replicate as well in HeLa cells [8,41,42].

In summary, we found that when inducing the invasive phenotype in *Salmonella*, dissimilarities—intrinsic and purposely altered—in growth media impact the degree of SPI-1 induction. These findings underscore the fact that the choice and makeup of culture media are as important as any other commonly controlled factors. In downstream utilization of SPI-1 induced bacteria, it is necessary to keep in mind how the bacteria have been grown and how that may shape experiment outcomes and analysis.
